# Breastfeeding and childhood asthma: a six-year population-based cohort study

**DOI:** 10.1186/1471-2431-7-39

**Published:** 2007-11-28

**Authors:** Pia Fredriksson, Niina Jaakkola, Jouni JK Jaakkola

**Affiliations:** 1 Environmental Epidemiology Unit, Department of Public Health, University of Helsinki, Helsinki, Finland; 2 Institute of Occupational and Environmental Medicine, The University of Birmingham, Birmingham, UK; 3 Department of Public Health Science and General Practice, University of Oulu, Oulu, Finland

## Abstract

**Background:**

The question of the protective effect of breastfeeding on development of asthma has raised substantial interest, but the scientific evidence of the optimal duration of breastfeeding is controversial.

**Methods:**

The authors elaborated the optimal duration of breastfeeding with respect to the risk of asthma primarily, and secondarily to the risk of persistent wheezing, cough and phlegm in school age in a population-based cohort study with the baseline in 1991 and follow-up in 1997. The study population comprised 1984 children aged 7 to 14 years at the end of the follow-up (follow-up rate 77). Information on breastfeeding was based on the baseline survey and information on the health outcomes at the follow-up.

**Results:**

There was a U-shaped relation between breastfeeding and the outcomes with the lowest risk with breastfeeding from four to nine months for asthma and seven to nine months for persistent wheezing, cough and phlegm.

**Conclusion:**

Our results suggest a U shape relation between duration of breastfeeding and risk of asthma with an optimal duration of 4 to 6 months. A true concave relation would explain the inconsistent results from the previous studies.

## Background

The question of the protective effect of breastfeeding on development of asthma has raised substantial interest, but the scientific evidence of the effects of breastfeeding is controversial [1,2]. Some epidemiologic studies provided evidence of a negative association consistent with protective effect [3-10], whereas some studies reported either no association [11-14], or a positive association between the duration of breastfeeding and the risk of asthma [15-19]. A strength of evidence indicates that short-term breastfeeding protects asthma up to 3–4 months [1,2].

Gdalevich and colleagues [2] conducted a systematic review and meta-analysis of all 12 prospective cohort studies, which fulfilled pre-stated inclusion criteria. The summary OR for the effect of exclusive breastfeeding for 3 months was 0.70 (95% CI 0.60, 0.81). Friedman and Zeiger [1] summarized in their recent review that exclusive breastfeeding should be encouraged for at least 4 to 6 months. However, there is little evidence of the role of long-term breastfeeding in the development of asthma.

We elaborated the relation of the duration of breastfeeding to the risk of asthma in a prospective cohort study of Finnish children. The wide range in the duration of breastfeeding in Finland allowed us to study breastfeeding over 12 months. We also studied the risk of persistent wheezing, cough and phlegm as secondary outcomes.

## Methods

### Study design

This was a prospective population-based cohort study to assess the relations between environmental exposures and other determinants and children's health.

### Study population and data collection

The source population comprised all children of the city of Espoo born between January 1, 1984, and December 31, 1989 [20]. Espoo is an urban-suburban municipality with a population of approximately 170 000 in 1990 and 200 000 in 2002. The baseline study population constituted of 2568 children between the ages of one to seven years, whose parents or guardians filled in a questionnaire (response rate 80.3%). In March 1997, we conducted a 6-year follow-up survey directed at all the members of the cohort. The home addresses of the participating children were updated by information from the Central Population Registry. A completed questionnaire was received from families of 1984 children (77.3% of the baseline study population). These children constituted the study population. The 6-year cohort did not differ substantially from the baseline study population, as shown in Table 1. The duration of breastfeeding was missing in 51 children and therefore the actual study population comprised 1933 children. A more detailed description of the questionnaire used in the baseline and the follow-up study can be found elsewhere [20]. The study was approved by the institutional ethics committee at the Department of Public Health, University of Helsinki.

**Table 1:** Comparison of the 6-year cohort and the baseline study population, Espoo, Finland 1991–1997.

	**Baseline**		**Lost to follow-up**		**6-year-cohort**	
**Characteristics at baseline**	**N**	**%**	**N**	**%**	**N**	**%**
Number	2568	100	584	22.7	1984	77.3
						
Age (years)						
1	424	16.5	100	17.1	324	16.3
2	405	15.8	104	17.8	301	15.2
3	310	16.0	92	15.8	318	16.0
4	400	15.6	67	11.5	333	16.8
5	415	16.2	101	17.3	314	15.8
6	391	15.2	81	13.9	310	15.6
7-	123	4.8	39	6.7	84	4.2

Gender						
Boy	1310	51.0	309	52.9	1001	50.5
Girl	1258	49.0	275	47.1	983	49.5

Any allergic disease						
Yes	337	13.1	81	13.9	256	12.9
No	2231	86.9	503	86.1	1728	87.1

Single parent/guardian						
Yes	183	7.1	53	9.1	130	6.6
No	2385	92.9	531	90.9	1854	93.4

Highest level of parental education						
Non-professional	498	19.5	129	22.3	369	18.7
Trade school	663	25.9	140	24.2	523	26.5
College or university	1395	54.6	310	53.5	1085	54.9
Missing information	12					

Exposure to ETS						
Yes	267	10.4	76	13.1	191	9.7
No	2291	89.6	504	86.9	1787	90.3
Missing Information	10					

Gas stove						
Yes	86	3.4	24	4.1	62	3.1
No	2469	96.6	556	95.9	1913	96.9
Missing Information	13					

Hairy/Feathery Pets						
Yes	480	18.7	113	19.3	367	18.5
No	2088	81.3	471	80.7	1617	81.5

Type of day care						
100% Home	940	36.6	210	36.0	730	36.8
100% Family	513	20.0	119	20.4	394	19.9
100% Centre	252	9.8	56	9.6	196	9.9
Combinations	863	33.6	199	34.1	664	33.5

### Definition and categories of breastfeeding

Information on duration of breastfeeding was based on the baseline data collection. Firstly a comparison of children with short and long duration of exclusive breastfeeding was performed. We studied the form of the relations using five *a priori *set categories: 0–3 months, 4–6 months, 7–9 months and 10–12 months and 12 months or longer. In the models we used the category with the lowest risk of the studied outcome as a reference category.

### Health outcomes

The primary outcome was asthma. Asthma was defined as doctor-diagnosed current asthma at the end of the follow-up period. The secondary outcomes included chronic respiratory symptoms: persistent wheezing, persistent cough, and persistent phlegm. Persistent wheezing was defined as wheezing apart from colds or wheezing most days or nights during the past year. Persistent cough was defined as a cough apart from colds for three months of the past year or more. Persistent phlegm was defined as phlegm production or dyspnea due to stuffiness in breathing apart from cold for three months during the past year or more.

### Covariates

To provide the best estimates of the relation between breastfeeding and the studied outcomes, several potential determinants of the outcome according to current knowledge were considered as potential confounders. Information on all the covariates used in the models was collected at baseline; some additional information was also collected in the follow-up survey. The following variables were included in the analyses: age, gender, parent's highest education, single parent or guardian, exposure to environmental tobacco smoke (ETS), smoking during pregnancy, parental atopy, and parental asthma, presence of hairy or feathery pets at home or type of day care during the past year. Parental asthma was defined as a doctor-diagnosed asthma in either or both of the child's parents. Parental atopy was defined as a doctor-diagnosed asthma or allergic rhinitis in either of the child's parents. Passive smoking was defined as someone in the household smoking inside the home during the past 12 months. Maternal smoking during pregnancy was included as a variable. This represented exposure to environmental tobacco smoke during the fetal period. Having pets in the household during pregnancy or during the lifetime of the child was controlled for. Type of day care was categorized into home, family care, day-care center or a combination of these. The children who had missing information on asthma either at baseline or follow-up survey were considered as not having asthma.

### Statistical methods

We compared the risk of the outcomes at the end of the follow-up in the five categories of breastfeeding. We used odds ratio as a measure of association between breastfeeding and the risk of the outcomes using the breastfeeding category with the lowest risk as a reference. Adjusted odds ratios were estimated applying logistic regression analysis. The odds ratios were adjusted for the covariates described earlier. We modeled upward and downward trends by fitting breastfeeding in months for selected ranges of breastfeeding based on the form of the breastfeeding-outcome relations.

## Results

### Follow-up and characteristic of the study population

The characteristics of the baseline population, those lost to follow-up and the 6-year cohort constituting the study population are presented in Table 1. Families with a single parent or smokers were over presented among those lost to follow-up. However, due to relatively high follow-up rate the study population differed only slightly from the baseline population.

The children with short breastfeeding were more often from a single-parent family, their parents were less educated, they were slightly more often at home care, and more commonly exposed to ETS and pets compared with the children with long breastfeeding (data not shown). These factors were taken into account in the multivariate analyses.

### Breastfeeding and the prevalence of asthma and chronic respiratory symptoms

**Figure 1:**
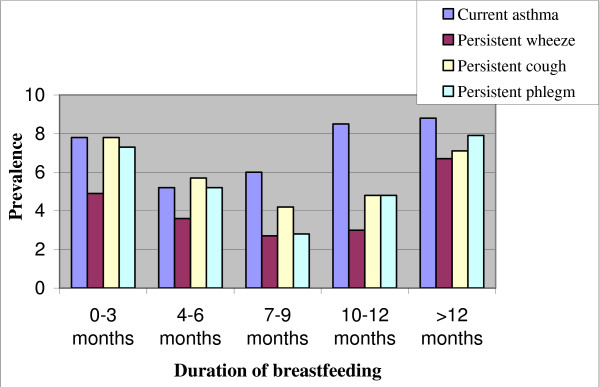
Prevalence of current asthma, persistent wheezing, cough and phlegm at the end of the follow-up period according to duration of breastfeeding.

Information on the duration of breastfeeding was missing for 51 children (2.6%) who were excluded from the study population. Only 1 child was not breastfed at all. Figure 1 illustrates U-shaped relations between the prevalence of the health outcomes and the duration of breastfeeding. The prevalence of asthma was at its lowest when a child was breastfed 4 to 6 months. The prevalence of persistent wheezing, cough and phlegm were at their lowest when a child was breastfed for 7 to 9 months (Table 2).

**Table 2:** Prevalence of current asthma, persistent wheezing, cough, and phlegm at the end of the follow-up period (N = 1933).

**Duration of breastfeeding**	**Current asthma**		**Persistent wheezing**		**Persistent cough**		**Persistent phlegm**	
	
	n *	**Prevalence (%)**	n *	**Prevalence (%)**	n *	**Prevalence (%)**	n *	**Prevalence (%)**
0–3 months (N = 346)	27	7.8	17	4.9	27	7.8	25	7.3
4–6 months (N = 385)	20	**5.2**	14	3.6	22	5.7	20	5.2
7–9 months (N = 528)	32	6.0	14	**2.7**	22	**4.2**	15	**2.8**
10–12 months (N = 434)	37	8.5	13	3.0	21	4.8	21	4.8
> 12 months (N = 240)	21	8.8	16	6.7	17	7.1	19	7.9

* Number of individuals with the health outcome

Table 3 presents the relations between the duration of breastfeeding and the risk of outcomes adjusted for potential confounding in logistic regression analysis. Adjusted ORs were calculated in two ways. First, short and long breastfeeding categories were contrasted to the reference category (4–6 months for asthma and 7–9 months for other health outcomes). Second, adjusted ORs of health outcomes were calculated for a one-month change in breastfeeding, either from optimal to shorter or to longer duration.

**Table 3:** The relation between the duration of breastfeeding and the risks of current asthma, persistent wheezing, cough, and phlegm at the end of the follow-up period (N = 1933).

**Duration of breastfeeding**	**Current asthma**		**Persistent wheezing**		**Persistent cough**		**Persistent phlegm**	
	
	Adjusted OR*	**95% CI**	Adjusted OR*	**95% CI**	Adjusted OR*	**95% CI**	Adjusted OR*	**95% CI**
0–3 months	1.44	0.78, 2.66	1.64	0.77, 3.52	1.72	0.94, 3.15	2.38	1.20, 4.70
4–6 months	1.00 (reference)		1.38	0.64, 2.97	1.35	0.73, 2.50	1.90	0.95, 3.79
7–9 months	1.16	0.65, 2.08	1.00 (reference)		1.00 (reference)		1.00 (reference)	
9–12 months	1.72	0.97, 2.08	1.15	0.53, 2.50	1.21	0.65, 2.25	1.87	0.95, 3.71
> 12 months	1.60	0.83, 2.08	2.26	1.07, 4.79	1.63	0.84, 3.16	2.88	1.42, 5.84
**Change in breastfeeding**								
From optimal to shorter per month ^†^	1.10	0.92, 1.32	1.12	0.99, 1.19	1.08	0.98, 1.19	1.11	1.00, 1.24
From optimal to longer per month ^‡^	1.03	1.00, 1.05	1.05	1.03, 1.08	1.04	1.02, 1.06	1.04	1.01, 1.06

* Adjusted in logistic regression analysis for age, gender, parent's highest education, single parent or guardian, exposure to environmental tobacco smoke (ETS), smoking during pregnancy, parental atopy, and parental asthma, presence of hairy or feathery pets at home or type of day care during the past year

^† ^Optimal was 7 months for asthma and 9 months for chronic respiratory symptoms

^‡ ^Optimal 7 months

The risk of asthma was elevated both when the child was breastfed for less than four months or more than six months. There was a statistically significant adjusted OR of 1.03 (95 % CI: 1.00, 1.05) per one-month increase in duration of breastfeeding. The adjusted odds ratio of asthma for one-month decrease in breastfeeding was 1.10 (95 % CI: 0.92, 1.32).

The risk of persistent wheezing increased 12 % per month from optimal to shorter duration and 5 % per month from optimal to longer duration of breastfeeding. The adjusted OR of wheezing for the contrast of over 12 months to 7–9 months was 2.26 (95 % CI: 1.07, 4.79). The risk of persistent cough increased 8 % per month from optimal to shorter duration and 4 % from optimal to longer duration. The risk of persistent phlegm increased 8 % and 4 % per month for decrease and increase in breastfeeding respectively.

## Discussion

The present population-based cohort study elaborated the relations between the duration of breastfeeding and the risk of asthma and chronic respiratory symptoms, such as wheezing, cough, and phlegm, which could be indicators of future asthma. The results illustrated U-shaped relations between breastfeeding and asthma, wheezing, and phlegm. The lowest prevalence of asthma was found in children who were breastfed from four to six months and of chronic respiratory symptoms when the child was breastfed from seven to nine months.

### Validity of results

A prospective cohort study offers a strong design to assess the relations between the duration of breastfeeding and the risk of asthma later in life. The follow-up rate (77%) in the present study was relatively high. The distributions of exposure indicators and the characteristics of the study population at baseline were similar and therefore losses to follow-up were not likely to introduce selection bias. The prospective study design minimizes information bias when information on the determinant of interest is collected before the onset of the studied outcome. Information on breastfeeding was collected mainly before the onset of the outcomes of interest. Only 52 of the asthma cases (27%) were present at the baseline which limits the possibility that any bias due to awareness of the disease or duration of breastfeeding was introduced. We were able to take into account most of the known potential confounders related to individual characteristics, and environmental exposures in the logistic regression analysis.

### Synthesis with previous knowledge

The epidemiological studies have provided controversial results showing negative [3-10], positive [15-19], and no association [11-14] between the duration of breastfeeding and the risk of asthma. Both methodological issues and the complexity of the phenomenon could explain controversial findings on the relation between breastfeeding and asthma. There are several factors, which may influence the direction and the quantity of association: the distribution of breastfeeding in the study population, the age range of disease experience, hereditary factors, environmental factors, and the modeling approach. Differences in these factors between different studies will provide heterogeneity in study-specific effect estimates for breastfeeding.

The distribution of the duration of breastfeeding in the study population is central. The range of duration varies substantially. In some study populations, including the U.S. [9] and New Zealand [19] a large proportion of children were not breastfed whereas in other populations, for example in Finland as shown in the present study, Norway [10], and Sweden [8], practically all children received breast milk and the median duration of breastfeeding is 5 to 7 months. This becomes critical when fitting a dichotomous variable if the true relation is non-linear, as suggested by the present study.

Another important issue is the duration of follow-up and the age of onset of asthma. If breastfeeding could delay the onset of asthma, the prevalence of current asthma would be lower among breastfed than non-breastfed in early age, but similar in later life. The meta-analysis of Gdalevich and colleagues [2] showed a stronger protective effect (summary OR = 0.47, 95 % CI 0.34, 0.66) in studies with less than a 2-year follow-up period compared to those with 2 or more years follow-up period (summary OR = 0.72, 95 % CI 0.62, 0.82). In the present study, we were interested in the long-term effects of breastfeeding and used the presence of asthma and respiratory symptoms at the age of 7–14 years as outcomes.

There is evidence that hereditary asthma or atopic diseases modifies the relation between duration of breastfeeding and the risk of asthma. The meta-analysis of Gdalevich [2] showed a protective effect of 3 months exclusive breastfeeding only in children of atopic parents (summary OR = 0.52, 95% CI 0.35, 0.79), whereas there was no effect in children of non-atopic parents (summary OR = 0.99, 95% CI 0.48, 2.03). In the present study, the concave relation between the duration of breastfeeding and the risk of asthma was similar both in children of non-atopic and atopic parents (data not shown).

Exposures to environmental factors could also modify the relation between breastfeeding and asthma. In a prospective cohort study in Oslo children, the effect of exposure ETS on the risk of lower respiratory tract infections [21] and asthma [10] were stronger among children who were breastfed less than 6 months compared those breastfed 6 months or longer. Similarly, a cross-sectional data from the third US National Health and Nutrition Examination Survey indicated that breastfeeding might reduce the prevalence of asthma and recurrent wheezing in children exposed to environmental tobacco smoke, but not in unexposed children [9]. In the present study, we adjusted for exposure to ETS and several other environmental factors, but did not elaborate second-degree effect modification.

A plausible explanation for controversial results in the previous studies is a true non-linear relation between the duration of breastfeeding and the risk of childhood asthma. An underlying non-linear relation could result in either negative or positive linear association depending on the distribution of breastfeeding length in the study population and the cut points and contrasts used in the analyses. Our results suggest a U-shaped relation, but do not exclude the possibility that different mechanisms of selection or confounding influence the results. A history of asthma in the family could influence the duration of breastfeeding and at the same time increase the risk of asthma in the child due to genetic effect and introduce a bias in attempts to assess the effect of breastfeeding on the risk of asthma. Duration of breastfeeding and the risk of asthma could have common determinants, which may cause confounding of the relation between breastfeeding and asthma. An example of a potential confounder is maternal smoking, which has been shown to be related to both a short duration of breastfeeding [22] and an increased risk of asthma [23].

## Conclusion

The original contribution of the present study is the evidence of a U-shaped rather than linear relation between duration of breastfeeding and the risk of asthma and chronic respiratory symptoms. A true concave relation would explain the inconsistent results from the previous studies using various cut-points for breastfeeding in the analyses. The results suggest that breastfeeding less than 4 months increases the risk of asthma and chronic respiratory symptoms. Also long breastfeeding was associated with an increased risk of the studied outcomes.

## Competing interests

The author(s) declare that they have no competing interests.

## Authors' contributions

PF contributed to data analyses, interpretation of the results and wrote the manuscript. NJ conducted the data management and contributed to the statistical analyses, interpretation of the results and writing of the paper. JJ designed and led the study, participated in the statistical analyses, and contributed to interpretation of the results and writing of the paper. All authors read and approved the final manuscript.

## Pre-publication history

The pre-publication history for this paper can be accessed here:


